# Assessing the impact of the Barbados sugar-sweetened beverage tax on beverage sales: an observational study

**DOI:** 10.1186/s12966-019-0776-7

**Published:** 2019-01-30

**Authors:** Miriam Alvarado, Nigel Unwin, Stephen J. Sharp, Ian Hambleton, Madhuvanti M. Murphy, T. Alafia Samuels, Marc Suhrcke, Jean Adams

**Affiliations:** 1 Centre for Diet and Activity Research, MRC Epidemiology Unit, University of Cambridge, Cambridge, UK; 2 George Alleyne Chronic Disease Research Centre, Caribbean Institute for Health Research, University of the West Indies, Bridgetown, Barbados; 3 Faculty of Medical Sciences, Cave Hill Campus, University of the West Indies, Bridgetown, Barbados; 4 Centre for Health Economics, University of York, York, UK

**Keywords:** Sugar-sweetened beverages, Evaluation, Fiscal policy, Obesity prevention, Diabetes prevention

## Abstract

**Background:**

The World Health Organization has advocated for sugar-sweetened beverage (SSB) taxes as part of a broader non-communicable disease prevention strategy, and these taxes have been recently introduced in a wide range of settings. However, much is still unknown about how SSB taxes operate in various contexts and as a result of different tax designs. In 2015, the Government of Barbados implemented a 10% ad valorem (value-based) tax on SSBs. It has been hypothesized that this tax structure may inadvertently encourage consumers to switch to cheaper sugary drinks. We aimed to assess whether and to what extent there has been a change in sales of SSBs following implementation of the SSB tax.

**Methods:**

We used electronic point of sale data from a major grocery store chain and applied an interrupted time series (ITS) design to assess grocery store SSB and non-SSB sales from January 2013 to October 2016. We controlled for the underlying time trend, seasonality, inflation, tourism and holidays. We conducted sensitivity analyses using a cross-country control (Trinidad and Tobago) and a within-country control (vinegar). We included a post-hoc stratification by price tertile to assess the extent to which consumers may switch to cheaper sugary drinks.

**Results:**

We found that average weekly sales of SSBs decreased by 4.3% (95%CI 3.6 to 4.9%) compared to expected sales without a tax, primarily driven by a decrease in carbonated SSBs sales of 3.6% (95%CI 2.9 to 4.4%). Sales of non-SSBs increased by 5.2% (95%CI 4.5 to 5.9%), with bottled water sales increasing by an average of 7.5% (95%CI 6.5 to 8.3%). The sensitivity analyses were consistent with the uncontrolled results. After stratifying by price, we found evidence of substitution to cheaper SSBs.

**Conclusions:**

This study suggests that the Barbados SSB tax was associated with decreased sales of SSBs in a major grocery store chain after controlling for underlying trends. This finding was robust to sensitivity analyses. We found evidence to suggest that consumers may have changed their behaviour in response to the tax by purchasing cheaper sugary drinks, in addition to substituting to untaxed products. This has important implications for the design of future SSB taxes.

**Electronic supplementary material:**

The online version of this article (10.1186/s12966-019-0776-7) contains supplementary material, which is available to authorized users.

## Background

In 2015, fifteen million people aged 30 and 70 years died from non-communicable diseases (NCDs) globally [1]. Despite the establishment of a target to reduce premature mortality from NCDs by one third, the WHO Independent High-Level Commission on NCDs has suggested that without a dramatic change in approach, this target will not be met [1]. A greater focus on population-level efforts to prevent NCDs is urgently needed [2].

The World Health Organization (WHO) identified 88 “Best Buys” to address the burden of NCDs [3]. One recommendation is to “reduce sugar consumption through effective taxation on sugar-sweetened beverages” [3]. Consumption of SSBs is associated with higher incidence of type 2 diabetes, overweight and obesity, cardiovascular risk factors, and dental caries [4–6]. Several countries have implemented or amended SSB taxes recently, including France, with a soft drinks tax of 7 cents/litre introduced in January 2012 (increased in July 2018); Mexico, with a specific 1 peso/litre tax since January 2014; Chile, with a tiered ad valorem tax since October 2014; the UK with a tiered levy incentivizing reformulation since April 2018, and several U.S. cities with taxes of 1–1.5 cents/ounce beginning in March 2015 [7–14]. The number of countries and localities implementing SSB taxes with a health focus has more than tripled since 2011 [7].

SSB taxes are hypothesized to increase the prices of SSBs, dampening demand and resulting in population-level improvement in health [15]. Evaluations of SSB taxes are beginning to emerge and provide some empirical evidence around these theoretical links. Prices of SSBs have been shown to increase following the implementation of an SSB tax [16–19]. Purchase of SSBs have been shown to decrease following the implementation of SSB taxes in Mexico and several U.S. cities and an amended SSB tax in Chile [16, 20–23]. Long-term health impacts have been estimated through modelling studies and have shown potential benefits [24, 25].

However, much is still unknown. SSB taxes can be designed in a wide variety of ways, and the specific design is likely to impact tax effectiveness [26]. Taxes can be structured as either specific, volume-based taxes (i.e. 1 peso/litre), sugar-content based taxes, or as ad valorem, value-based taxes (i.e. 10% of the manufacturer’s price). It has been suggested (but not shown) that ad valorem SSB taxes may encourage brand down-switching, the consumer strategy of substituting to cheaper brands, since taxing drinks proportionate to their value may create a steeper price gradient amongst diverse products [26, 27]. This may undermine some of the intended health benefits of an SSB tax by incentivizing behaviors that do not necessarily reduce sugar consumption [26]. Out of 34 countries with SSB taxes (according to the 2018 NOURISHING report), 11 rely on a purely ad valorem tax structure and two use a mixed ad valorem and specific rate tax structure [8]. Assessments of global tobacco taxes have highlighted that ad valorem structures tend to be favored by low-income countries, and there is early evidence that this pattern applies to SSB taxation as well [8, 28]. Understanding the impact of these different tax structures on consumer behavior will be critical in enabling policymakers to design taxes that are most likely to be effective from a health perspective, and to ensure that differences between SSB tax structures in low and high-income countries do not exacerbate health inequalities.

### The Barbados SSB tax

According to a nationally representative survey carried out in Barbados from 2012 to 2013, the prevalence of obesity amongst adults 25 and older was 33.8% (compared to global estimates of obesity prevalence of 10 and 14% for men and women), and the prevalence of diabetes was 18.7% (compared to a global average of 8.3%) [29]. In June 2015, the Government of Barbados announced the introduction of a 10% ad valorem tax on SSBs to address the high burden of non-communicable diseases in Barbados [30]. Taxable products included “sweetened beverages such as carbonated soft drinks, juice drinks, sports drinks, fruit juices […] that contain added high calorie sweeteners” [30]. Bottled waters, 100% juices, coconut water, unsweetened milk and powdered drinks were exempt. The tax was structured as an ad valorem tax, mirroring the tax structure recommended by the International Monetary Fund (IMF) in a 2014 report to the Government of Barbados [31]. Initial analyses of price changes following the Barbados tax suggest that SSB prices increased by 5.9%, while prices of non-SSBs remained constant [18].

We aimed to assess whether and to what extent there was a change in sales of SSBs following implementation of the Barbados SSB tax. In addition, we evaluated whether the ad valorem tax structure was associated with brand down-switching, as hypothesized but not empirically examined to date.

## Methods

We used electronic point of sale data from a major grocery store chain. We utilized an interrupted time series (ITS) design, controlling for seasonality, autocorrelation, and other time-varying factors such as tourism and inflation [32]. To address concerns around time-varying confounding, we conducted sensitivity analyses with two control groups. We included a post-hoc stratification by price tertile to assess the extent to which consumers engage in brand down-switching.

### Setting

Barbados is a small island developing state (SIDS) in the Eastern Caribbean. Recent nationally representative surveys have found high rates of SSB consumption, overweight, obesity, and type 2 diabetes in the adult population [29].

### Data

Electronic point-of-sale data were available from a major grocery chain in Barbados, representing 32% of the grocery store market share in Barbados (personal communication). Data were provided aggregated across all individual stores. We regrettably were not able to access information about shopper demographics. The primary outcome was sales (measured in volume) of SSBs and non-SSBs, as defined by the tax policy. Dairy beverages were not included due to data availability. Sub-category analyses were conducted on carbonated SSBs, other SSBs (including sweetened juice drinks), waters and other non-SSBs (including no-added sugar (NAS) juices). All products were categorized according to the definitions in Additional file 1: Table S1. Juices were initially categorized according to product descriptions (i.e. “100% juice” and “NAS juice” were identified as unsweetened juices). These categorizations were further refined based on a manual search for product-specific ingredients listed on manufacturer’s websites and in-store observations of nutrient panels. Products with no available size data were excluded from the analysis (39 products, accounting for 1.7% of total products sold). Data were available from January 1, 2013-October 31, 2016, with dollar and unit sales aggregated by week (1161 unique, size-specific beverage products, no missing weeks). This study period includes 141 weeks of pre-intervention data and 59 weeks post-tax.

### Analysis

We used an interrupted time series (ITS) design (uncontrolled and controlled) to assess trends in sales of SSBs, non-SSBs, and beverage sub-categories. To address some of the major threats to validity associated with ITS designs, we were guided by the checklist for quality criteria (see Additional file 1: Box 1) [33].

#### Overall change in sales

We calculated the weekly volume in millitres sold per capita for SSBs and non-SSBs, as well as for carbonated-SSBs, other SSBs, water and other non-SSBs. We used ordinary least square regression assuming a normally distributed outcome, and built our models using an interrupted time series design to estimate change in sales following tax implementation. We included both an intercept effect (an indicator denoting the post-tax period) and a trend effect (zero in the pre-tax period and 1 in the week the tax was implemented, 2 for the second week of taxation and so on). Previous analyses of SSB sales following tax implementation have found both immediate step changes and changes in trend, so we allowed for both [16, 22]. We also included an overall linear trend effect (1 to 200) to account for the pre-tax linear trend in beverage sales.

We included monthly indicators (1-11) to allow the seasonal effect to be modelled with maximum flexibility. To account for other underlying trends, we included two likely important time-varying covariates: monthly tourist arrivals (to control for changing demand driven by tourism) and monthly consumer price index (to control for inflation). We present the absolute and relative difference from the counterfactual (setting the variables for the post-tax period and post-tax trend to zero). See Additional file 1: Text 1 for further details.

To assess model goodness of fit, we examined the model residuals to assess whether they were normally distributed, and whether they were randomly distributed over time. We tested for autocorrelation using the Cumby-Huizinga test and included a single lag of the residual which adequately addressed autocorrelation.

#### Sensitivity analyses

To address potential concerns related to time-varying confounding, we conducted two sensitivity analyses. The first sensitivity analysis was an assessment of the same outcome (drink sales) in a setting without an SSB tax (Trinidad and Tobago), and the second was an assessment of a different outcome (vinegar sales) in Barbados. Further details are provided in Additional file 1: Text 2.

#### Change in sales by price tertile

We divided each beverage category into three price levels (low- mid- and high-cost) based on the average price observed across the study period, and repeated the main analysis by price tertile (see Additional file 1: Text 3).

All analyses were conducted using STATA v14.2 [34].

## Results

### Overall change in sales

Sales of SSBs were lower than predicted over the 59-week post-tax period. On average, sales changed by − 8.6 mL/capita/week [95% CI -10.0 to − 7.3] compared to the counterfactual, equivalent to a − 4.3% [− 4.9 to − 3.6%] change relative to the counterfactual. Sales of carbonated SSBs decreased, both overall and at the end of the study period. Sales of other SSBs decreased overall, although at the end of the study period there was no evidence of a statistically significant difference.

Sales of non-SSBs were higher in the post-tax period than predicted, with an average increase of 6.1 mL/capita/week [5.3 to 6.8], equivalent to a 5.2% [4.5 to 5.9%] relative change. Sales of bottled water increased, both overall and at the end of the study period. Sales of other non-SSBs increased overall, although at the end of the study period there was no evidence of a difference. The residuals across all models did not indicate any violation of the model assumptions.

**Fig. 1:**
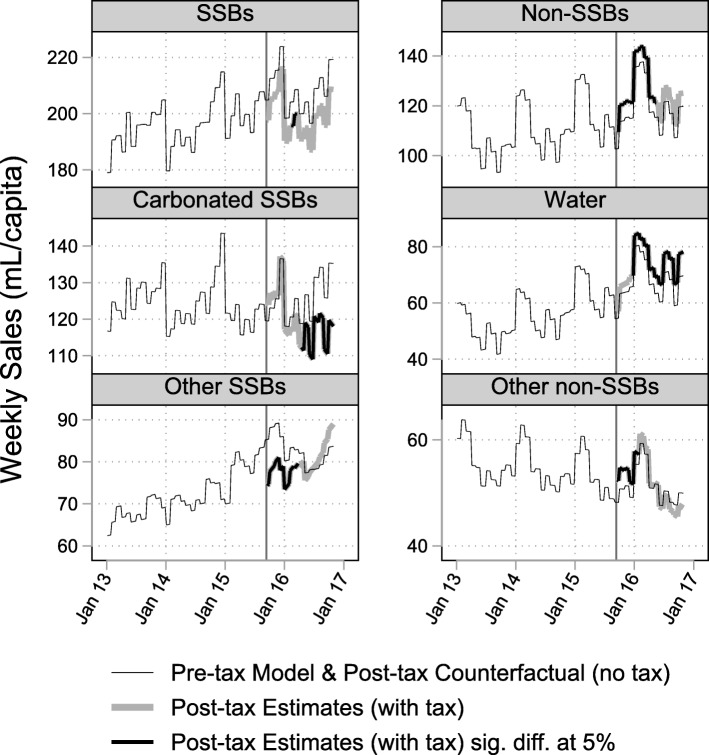
Grocery Store Sales (mL/capita/week), January 2013–October 2016

See Fig. 1, Table 1 and Additional file 1: Table S3 for detailed results.

**Table 1:** Mean post-tax absolute and relative effects, overall and in the final study week

	Mean Overall				Final Week of Study			
	Absolute (mL/capita/week)		Relative (%)		Absolute (mL/capita/week)		Relative (%)	
	Est	CI	Est	CI	Est	CI	Est	CI
SSBs	−8.6	− 10.0 to − 7.3	− 4.3	− 4.9 to − 3.6	− 10.4	− 26.8 to 6.0	− 5.9	− 15.5 to 3.7
*Carbonated SSBs*	− 4.5	− 5.4 to − 3.6	− 3.6	− 4.4 to − 2.9	−15.6	− 26.8 to − 4.5	−15.5	−27.4 to − 3.7
*Other SSBs*	−4.1	− 4.6 to − 3.6	−5.1	− 5.8 to − 4.5	4.1	−2.2 to 10.5	5.1	−2.6 to 12.8
Non-SSBs	6.1	5.3 to 6.8	5.2	4.5 to 5.9	5.4	−3.8 to 14.6	3.8	−2.7 to 10.2
*Water*	4.9	4.3 to 5.5	7.5	6.5 to 8.3	8.1	1.1 to 15.0	9.1	1.5 to 16.8
*Other non-SSBs*	1.3	1.0 to 1.6	2.4	1.9 to 3.1	−2.3	−5.9 to 1.3	−4.3	−11.1 to 2.5

### Sensitivity analyses

The overall pattern of results was robust to both sensitivity analyses (see Additional file 1: Tables S4-S5). In the model that controlled for country, average sales of SSBs changed by − 8.2 mL/capita/week [95% CI -9.5 to − 7.0] in Barbados compared to a − 0.4 mL/capita/week [95% CI -1.8 to 0.9] in Trinidad and Tobago (with no SSB tax). In the model that controlled for a different grocery store item, average sales of SSBs changed by − 8.0 mL/capita/week [95% CI -9.2 to − 6.8] compared to 2.6 mL/capita/week [95% CI 1.4 to 3.8] for vinegar.

### Change in sales by price tertile

**Fig. 2:**
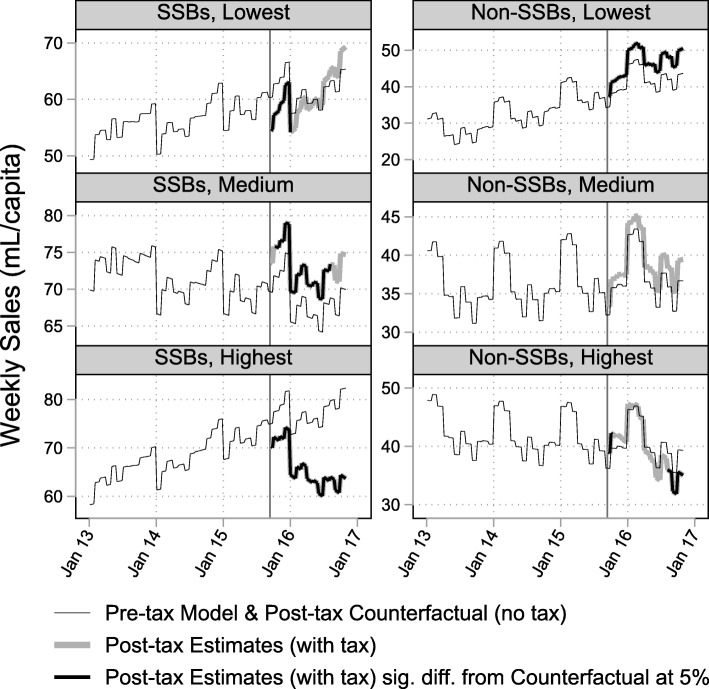
Volume of SSBs and non-SSBS sold in Barbados, by price tertile January 2013-October 2016

**Fig. 3:**
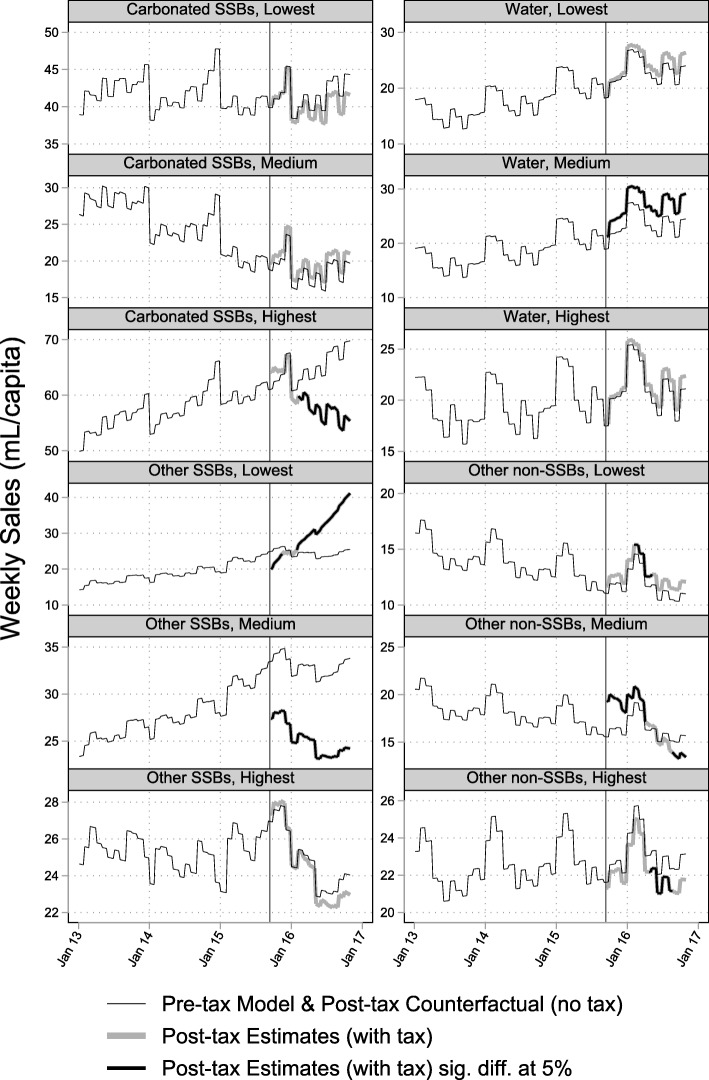
Volume of product sub-categories sold in Barbados, by price tertile January 2013-October 2016

Table 2 and Figs. 2-3 summarize the results of the post-hoc price tertile stratification. Sales of low-cost SSBs decreased immediately following the tax, before returning to predicted levels. Sales of mid-range SSBs increased, while sales of high-cost SSBs decreased across the whole study period. The differences in trends between low-cost and mid−/high-cost tertiles were statistically significant at the 5% level (see Additional file 1: Table S7).

**Table 2:** Mean post-tax absolute and relative effects, by price tertile

		Mean Overall				Final Week of Study			
		Absolute (mL/capita)		Relative (%)		Absolute (mL/capita/week)		Relative (%)	
	Level	Est	CI	Est	CI	Est	CI	Est	CI
SSBs	Low-cost	−1.5	−2.0 to − 1.0	− 2.6	−3.4 to − 1.7	3.3	−2.0 to 8.5	5.5	−3.2 to 14.1
	Mid-range	4.3	3.8 to 4.8	6.4	5.6 to 7.2	4.7	−0.6 to 9.9	7.1	− 0.7 to 14.8
	High-cost	−10.9	−11.4 to − 10.4	−14.4	− 15.1 to − 13.8	−17.7	−23.0 to − 12.4	−31.8	−42.7 to − 21.0
*Carbonated SSBS*	Low-cost	− 0.9	−1.3 to − 0.6	− 2.3	− 3.3 to − 1.4	− 2.5	− 6.7 to 1.7	− 7.1	− 19.2 to 5.1
	Mid-range	1.2	0.8 to 1.6	6.5	4.2 to 8.6	1.3	−2.9 to 5.5	8.6	− 18.3 to 35.6
	High-cost	−4.6	−5.0 to − 4.2	− 7.2	− 7.8 to − 6.5	− 13.2	− 17.4 to − 9.0	−26.3	−35.7 to − 17.0
*Other SSBs*	Low-cost	4.2	3.9 to 4.4	17.6	16.4 to 18.6	14.2	11.6 to 16.8	33.0	27.6 to 38.4
	Mid-range	−7.6	−7.9 to − 7.4	−23.7	−24.5 to − 22.9	−9.4	−12.0 to −6.8	−33.7	−44.5 to − 22.9
	High-cost	−0.2	− 0.5 to 0.0	− 0.9	−2.0 to 0.1	−1.0	− 3.6 to 1.6	− 3.6	−13.6 to 6.3
Non-SSBs	Low-cost	4.7	4.4 to 5.0	11.3	10.6 to 12.1	6.6	3.3 to 9.8	11.9	6.3 to 17.4
	Mid-range	1.8	1.5 to 2.1	4.9	4.1 to 5.8	2.7	−0.5 to 5.9	5.9	−1.1 to 13.0
	High-cost	−0.3	−0.6 to − 0.0	−0.9	−1.6 to − 0.1	−3.8	−7.0 to − 0.6	−9.2	−17.4 to − 1.1
*Water*	Low-cost	1.1	0.9 to 1.4	5.1	4.1 to 6.2	2.2	−0.3 to 4.7	7.5	−0.8 to 15.9
	Mid-range	3.3	3.1 to 3.5	14.5	13.5 to 15.5	4.5	2.0 to 7.0	14.1	6.6 to 21.5
	High-cost	0.6	0.4 to 0.9	3.1	1.8 to 4.2	1.2	−1.3 to 3.7	4.7	−5.1 to 14.4
*Other non-SSBs*	Low-cost	0.8	0.7 to 0.9	6.6	5.6 to 7.7	0.9	−0.4 to 2.3	6.5	−2.5 to 15.4
	Mid-range	1.1	1.0 to 1.2	6.7	5.9 to 7.5	−1.8	−3.2 to −0.5	−11.0	− 19.6 to − 2.4
	High-cost	− 1.3	−1.4 to − 1.2	−5.7	−6.3 to − 5.2	− 2.7	−4.1 to − 1.4	−12.0	− 18.3 to − 5.8

Low-cost non-SSBs increased across the whole study period, while there was no sustained evidence of a change in sales of mid-range non-SSBs. Sales of high-cost non-SSBs increased immediately after the tax and decreased below predicted levels by the end of the study period. The difference in trends between price tertiles were all statistically significant.

The product sub-category analysis showed a similar overall pattern of results. High-cost carbonated-SSBs decreased, and low-cost other SSBs increased while mid-range other SSBs decreased. Mid-range bottled water sales increased, and mid-range other non-SSBs increased immediately before decreasing by the end of the study period. High-cost other non-SSBs decreased. See Figs. 2-3, Table 2 and Additional file 1: Tables S6-S7 for detailed results.

Coefficients from all models are summarized in Additional file 1: Table S8.

## Discussion

In this first analysis of the impact of an SSB tax in a small island developing state, we found that the implementation of a 10% ad valorem tax was associated with a 4.3% decrease in grocery store sales of SSBs and 5.2% increase in sales of non-SSBs. Sensitivity analyses using a cross-country control and a within-country control led to a similar pattern of results.

We stratified by price tertile and found evidence of brand down-switching, with sales of expensive SSBs decreasing by 7.2% and sales of mid-range SSBs increasing by 6.5% To our knowledge, this is the first evaluation of a de novo ad valorem tax, and the first evaluation to explicitly test for differential effects by baseline price range.

### Strengths and weaknesses of the study

We used electronic point of sale data from a major grocery store chain. These data may not be representative of all SSB sales and the findings from these data are limited to purchasing behaviors amongst the subset of people who shop at this chain. It is possible that consumers may have shifted to another store following the tax, which could mean our estimate of effect may be exaggerated. However, we assessed both non-SSB sales and vinegar sales and found no evidence of a decrease in these untaxed products, supporting the hypothesis that the tax did not lead consumers to change stores. We did not assess dairy, powdered drinks, concentrates or syrups used to make drinks, nor did we estimate potential substitution to other non-beverage products such as high-sugar confectionary. Unlike analyses using household purchase data, we relied on aggregated weekly sales data and thus were not able to conduct sub-group analyses that would have allowed a stratification by socioeconomic status [22, 35, 36]. Because we used sales, not purchase data we were not able to estimate absolute changes in terms of mL purchased per person as has been done elsewhere [20–22].

Despite limitations, these data were the most detailed source available in this setting. Similar data have been used in other SSB evaluations in the US [16]. There is no commercial purchase panel available in Barbados, and this is likely to be the case in many other SIDS or low/middle income settings. In addition, commercial purchase panel data are very expensive, and alternative data sources may be important for assessing policy evaluations in a range of contexts. Commercial panel data rely on accurate reporting by the heads of household, may systematically underestimate on-the-go purchases and only capture purchases made by urban households. Repeated cross-sectional surveys represent an additional type of data that have been used in SSB tax evaluations, although these surveys may be biased by social desirability particularly after advocacy around SSB taxation [16, 35].

In contrast, electronic point of sale data do not rely on individual reporting and can provide a rich, consistently measured time series. We had a long and balanced time series which has been shown to add strength to the ITS design [37]. Since Barbados is a relatively isolated island, the risk of cross-border shopping was virtually zero, in contrast to some of the U.S. evaluations of city-specific SSB taxes [16, 38].

One major challenge with the ITS designs is the potential for time-varying confounding, i.e. the possibility that other events or policies occurred concurrently with the intervention, which may influence the outcome. To address this, we used a control group in the same population (vinegar), and a control group using the same outcome in a population without an SSB tax (Trinidad and Tobago) to increase the internal validity of the study [35, 39]. While it is a major challenge to find the ‘perfect’ country comparator, Barbados and Trinidad and Tobago have been estimated to have similar levels of SSB consumption and share demographic and cultural characteristics (Additional file 1: Table S2). Although it is not possible to make causal claims following this type of observational study, we find compelling evidence that the Barbados SSB tax was associated with changes in sales of SSBs and non-SSBs, after controlling for time-varying factors and underlying trends. Our previous finding that prices of SSBs increased following implementation of the tax also strengthens the hypothesis that the tax was associated with these observed changes in sales.

### In relation to other studies

An evaluation of the Mexico SSB tax (a specific tax of 1 peso/litre) found a 6.0% decrease in SSB purchases in the first year following the tax [22]. This effect was driven by a large reduction in purchases of non-carbonated SSBs (17%), alongside a much smaller reduction in purchases of carbonated SSBs (1.2%) [22]. In contrast, we found that the reduction in SSB sales in Barbados was driven by a persistent reduction in carbonated SSBs, with a 15.5% reduction in carbonated SSB sales by the end of the study period. These differences may be explained by variations in the beverage market, tax structure, price changes, and other changes concurrent with tax implementation. The Mexico SSB tax was more fully passed on than the Barbados tax, consistent with variation in the respective tax structures. The implementation of the Mexico tax was associated with, on one hand intense industry marketing and promotions, and on the other hand health campaigns about SSBs and a larger government-led focus on obesity control and prevention [22, 40]. These concurrent changes may have contributed to shifting norms around SSBs, independent of the price effect of the tax. In Barbados, no coordinated national health campaigns were introduced alongside the tax (personal communication) [41]. The extent to which the tax influenced media representations of SSBs is currently under investigation.

Evaluations of the amended SSB tax in Chile (an ad valorem tax modified from a single rate of 13% for all SSBs to two tiers: 10% for low-sugar SSBS and 18% for high-sugar SSBs) found mixed evidence. One evaluation estimated a 3.4% reduction in purchases of high-sugar SSBs and a 10.7% increase in low-sugar SSBs [21]. Amongst the high-sugar SSBs, the reduction was driven by non-carbonated SSBs (8.2%), with no statistically significant overall change observed amongst carbonated SSBs. This pattern was similar to findings from Mexico, but again contrasted with our findings around reductions in carbonated SSBs. Another evaluation found a 21.6% reduction in purchases of high-sugar SSBs and no statistically significant change in low-sugar drinks [20].

Several evaluations have been conducted in U.S. cities following implementation of SSB taxes. An evaluation of the Berkeley, California SSB tax (a specific tax of 1 cent/oz) used electronic point of sale data, as we did, and included data from stores in untaxed locations. They found a decrease of 9.6% in SSB sales concurrent with a 6.9% increase in sales in untaxed locations, suggesting that consumers may have engaged in cross-border shopping and that the estimated decrease may have been exaggerated. We found a 4.3% decrease in SSB sales in Barbados, and no change in untaxed locations (Trinidad and Tobago), consistent with the hypothesis that cross-border shopping would be negligible in a SIDS context.

An evaluation of the Mexico tax found an increase in non-SSB purchases of 4.0% in the first year, primarily driven by increases in bottled water purchases [22]. Sales of non-SSBs increased by 3.5% in Berkeley, driven primarily by increased in bottled water sales (15.6%). Household purchases of non-SSBs in Chile were found to either decrease (3.1%) or remain unchanged following the tax modification, perhaps due to lower-sugar SSBs becoming relatively cheaper after the tax amendment (going from a tax rate of 13% to 10%) and crowding out untaxed beverages [21]. Our findings were similar to these other studies, with the exception of the Chile evaluations. None of the existing SSB tax evaluations assessed potential brand down-switching.

### Meaning of the study

This study suggests that the Barbados SSB tax was effective at reducing sales of SSBs and increasing sales of non-SSBs in a major grocery store chain. An exploratory analysis suggests that brand-down switching may have led to an increase in sales of cheaper SSBs. Brand down-switching has been observed following tobacco taxation and is thought to be of particular concern with ad valorem (value-based) taxes in general [26, 35, 42]. This evaluation provides initial evidence that brand down-switching may also occur following an ad valorem SSB tax. This has important consequences from a health perspective and may reduce the potential public health effectiveness of SSB taxes. A policy that encourages consumers to substitute towards cheaper SSBs may lead to an increase in sugar consumption in cases where cheaper SSBs are associated with higher levels of sugar. On the other hand, if cheaper SSBs are lower in sugar content (such as sugar-sweetened flavored waters) overall sugar consumption may still be reduced.

It is important to evaluate ad valorem SSB taxes (in addition to specific SSB taxes), to assess whether tax structure is associated with a differential effect. An increasing number of countries have implemented ad valorem SSB taxes, including Peru (25%), the United Arab Emirates (50%) and Chile (18%) [7, 8]. However, if ad valorem taxes incentivize brand down-switching more than specific taxes, they may undermine some of the intended health impact of these policies. If, as has been demonstrated in a global assessment of tobacco taxation policy, low-income countries are more likely to implement ad valorem tax structures than their high-income counterparts, this could have important consequences from a global health equity perspective [28].

### Future research

Sugar-sweetened beverage taxes are a relatively new policy instrument, and there is much to be learned about how these taxes function. First, it will be helpful to develop a better understanding of the extent to which consumers substitute to cheaper alternatives (such as other SSBs or non-beverage high-sugar confectionary, rather than to non-SSBs), and to also measure the impact this may have on total sugar consumption. Second, it will be necessary to assess the extent to which behavioral responses vary by socioeconomic group and by gender and age. Third, it will be useful to model demand systems to simulate a range of future policy options, including increased SSB tax rates, and taxes or subsidies on other food and beverages. It has been suggested that SSB taxes should be structured to increase the price of SSBs by 20% or more (while the 10% Barbados tax was associated with a 5.9% price increase) [18, 26, 43]. Future simulation studies should take into account tax structure as well, and explicitly address potential brand down-switching behaviors.

Finally, it will be important to continue to elaborate on and build a unified theory around how SSB taxes operate. Currently most evaluations test the implicit hypothesis that price change drives change in SSB consumption [15]. However, SSB taxes exist in complex and adaptive systems, and it is likely that they (like tobacco taxes) may operate in more complex ways [35]. SSB taxes have been implemented in a diverse range of settings (i.e. low vs. high baseline SSB consumption), among populations with differing levels of disposable income and at very different levels (national, local). Additional evaluations are needed to help illustrate the potential mediating effect that these contextual factors may have on tax effectiveness, and to help guide the generalizability of these evaluation studies.

## Conclusions

We find evidence that the Barbados SSB tax was associated with a reduction in sales of SSBs, after controlling for underlying trends. This finding was robust to sensitivity analyses using a within-country control (vinegar) and a cross-country control (Trinidad and Tobago). Brand down-switching may have led to an increase in the sales of some low- and mid-cost SSBs. A continued assessment of the Barbados SSB tax will be helpful, in particular to further develop theory around SSB tax mechanisms and to explore possible substitution effects and the associated impact of these substitutions on total dietary intake.

## Additional file

Additional file 1: A description of additional methods, descriptive data, sensitivity analyses and more detailed results tables and figures (DOCX 70 kb)

## Abbreviations

NCD: Non-communicable disease
Non-SSB: Non-sugar-sweetened beverage
SIDS: Small island developing state
SSB: Sugar-sweetened beverage
WHO: World Health Organization

## References

[1] Nishtar S, Niinistö S, Sirisena M, Vázquez T, Skvortsova V, Rubinstein A (2018). Time to deliver: report of the WHO independent high-level commission on NCDs. Lancet.

[2] Horton R, Sargent J (2018). 2018 must be the year for action against NCDs. Lancet Lond Engl.

[3] World Health Organization. Tackling NCDs:‘best buys’ and other recommended interventions for the prevention and control of noncommunicable. diseases. 2017.

[4] Malik VS, Popkin BM, Bray GA, Despres J-P, Willett WC, Hu FB (2010). Sugar-sweetened beverages and risk of metabolic syndrome and type 2 diabetes: a meta-analysis. Diabetes Care.

[5] Imamura F, O’Connor L, Ye Z, Mursu J, Hayashino Y, Bhupathiraju SN (2016). Consumption of sugar sweetened beverages, artificially sweetened beverages, and fruit juice and incidence of type 2 diabetes: systematic review, meta-analysis, and estimation of population attributable fraction. Br J Sports Med.

[6] Tedstone A, Targett V, Allen R, Staff at PHE. Sugar Reduction: The evidence for action [Internet]. London, UK: Public Health England; 2015 Oct [cited 2018 Dec 17]. Available from: https://assets.publishing.service.gov.uk/government/uploads/system/uploads/attachment_data/file/470179/Sugar_reduction_The_evidence_for_action.pdf

[7] Smith E, Scarborough P, Rayner M, Briggs ADM (2018). Should we tax unhealthy food and drink?. Proc Nutr Soc.

[8] World Cancer Research Fund International. NOURISHING: Use economic tools to address food affordability and purchase incentives [Internet]. [cited 2018 Nov 9]. Available from: https://www.wcrf.org/sites/default/files/Use-economic-tools.pdf

[9] City of Berkeley. SSB Tax Ordinance 7,388-N.S. [Internet]. Dec 18, 2014. Available from: https://www.cityofberkeley.info/uploadedFiles/Health_Human_Services/Level_3_-_Public_Health/SSB%20Tax%20Ordinance%207,388-N.S..pdf.

[10] Great Britain, Treasury, Gauke D. Budget 2016: return to an order of the house of commons dated 16 march 2016 : copy of the budget report - march 2016 as laid before the house of commons by the chancellor of the Exchequer when opening the budget. 2016.

[11] Secretaría de Salud. Estrategia Nacional para la Prevención y el Control del Sobrepeso, la Obesidad y la Diabetes Mexico: IEPSA, Entidad Paraestatal del Gobierno Federal; 2013.

[12] Ministerio del Interior y Seguridad Publica Diario Oficial de la Republica de Chile. Num 40.969. 2014 Sep 29 [cited 2018 Dec 18]; Available from: http://www.diariooficial.interior.gob.cl/media/2014/09/29/do-20140929.pdf

[13] LOI n° 2011-1977: CONDITIONS GÉNÉRALES DE L’ÉQUILIBRE FINANCIER [Internet]. Dec 28, 2011. Available from: https://www.legifrance.gouv.fr/eli/loi/2011/12/28/BCRX1125684L/jo/texte

[14] Council President Clarke, Councilmember Henon. Amending Title 19 of The Philadelphia Code, entitled “Finance, Taxes and Collections,” by adding a new Chapter 19–4100, entitled “Sugar-Sweetened Beverage Tax,” under certain terms and conditions. [Internet]. 160176 Jun 16, 2016. Available from: https://phila.legistar.com/LegislationDetail.aspx?ID=2595907&GUID=36060B21-D7EE-4D50-93E7-8D2109D47ED1&FullText=1

[15] Mytton OT, Eyles H, Ogilvie D (2014). Evaluating the health impacts of food and beverage taxes. Curr Obes Rep.

[16] Silver LD, Ng SW, Ryan-Ibarra S, Taillie LS, Induni M, Miles DR (2017). Changes in prices, sales, consumer spending, and beverage consumption one year after a tax on sugar-sweetened beverages in Berkeley, California, US: a before-and-after study. PLoS Med.

[17] Colchero MA, Salgado JC, Unar-Munguía M, Molina M, Ng S, Rivera-Dommarco JA (2015). Changes in prices after an excise tax to sweetened sugar beverages was implemented in Mexico: evidence from urban areas Nugent RA, editor. PLOS ONE.

[18] Alvarado M, Kostova D, Suhrcke M, Hambleton I, Hassell T, Samuels TA (2017). Trends in beverage prices following the introduction of a tax on sugar-sweetened beverages in Barbados. Prev Med.

[19] Falbe J, Rojas N, Grummon AH, Madsen KA (2015). Higher retail prices of sugar-sweetened beverages 3 months after implementation of an excise tax in Berkeley, California. Am J Public Health.

[20] Nakamura R, Mirelman AJ, Cuadrado C, Silva-Illanes N, Dunstan J, Suhrcke M (2018). Evaluating the 2014 sugar-sweetened beverage tax in Chile: an observational study in urban areas. PLoS Med.

[21] Caro JC, Corvalán C, Reyes M, Silva A, Popkin B, Taillie LS (2018). Chile’s 2014 sugar-sweetened beverage tax and changes in prices and purchases of sugar-sweetened beverages: an observational study in an urban environment. PLoS Med.

[22] Colchero MA, Popkin BM, Rivera JA, Ng SW. Beverage purchases from stores in Mexico under the excise tax on sugar sweetened beverages: observational study. BMJ. 2016:h6704. 10.1136/bmj.h6704 PMC4986313 26738745

[23] Falbe J, Thompson HR, Becker CM, Rojas N, McCulloch CE, Madsen KA (2016). Impact of the Berkeley excise tax on sugar-sweetened beverage consumption. Am J Public Health.

[24] Eyles H, Mhurchu CN, Nghiem N, Blakely T (2012). Food pricing strategies, population diets, and non-communicable disease: a systematic review of simulation studies. PLoS Med.

[25] Sánchez-Romero LM, Penko J, Coxson PG, Fernández A, Mason A, Moran AE (2016). Projected impact of Mexico’s sugar-sweetened beverage tax policy on diabetes and cardiovascular disease: a modeling study. PLoS Med.

[26] Waqanivalu T, Nederveen L, World Health Organization. Fiscal policies for diet and prevention of noncommunicable diseases: technical meeting report, 5-6 May 2015, Geneva, Switzerland. [Internet]. 2016 [cited 2017 Apr 20]. Available from: http://apps.who.int/iris/bitstream/10665/250131/1/9789241511247-eng.pdf

[27] Brownell KD, Farley T, Willett WC, Popkin BM, Chaloupka FJ, Thompson JW (2009). The public health and economic benefits of taxing sugar-sweetened beverages. N Engl J Med.

[28] Organization WH. WHO technical manual on tobacco tax administration: World Health Organization; 2010. 136 p

[29] Howitt C, Hambleton IR, Rose AMC, Hennis A, Samuels TA, George KS (2015). Social distribution of diabetes, hypertension and related risk factors in Barbados: a cross-sectional study. BMJ Open.

[30] Sinckler C. Presentation of the Financial Statement and Budgetary Proposals 2015 [Internet]. 2015 Jun [cited 2017 May 25]. Available from: https://www.barbadosparliament.com/uploads/document/d1efb84aac6a7abe4c6c0efcf8ceedd2.pdf

[31] Krelove R, Crivelli E, Gendron P-P. Barbados: a tax reform roadmap for simplicity and revenue buoyancy. IMF; 2014.

[32] Hawkes C, Alderman H, Chaloupka F, Harris J, Kumanyika S, Smed S (2017). Principles behind evaluations of national food and beverage taxes and other regulatory efforts: letter to the editor. Obes Rev.

[33] Ramsay CR, Matowe L, Grilli R, Grimshaw JM, Thomas RE (2003). Interrupted time series designs in health technology assessment: lessons from two systematic reviews of behavior change strategies. Int J Technol Assess Health Care.

[34] StataCorp. Stata Statistical Software: Release 14. College Station, TX: StataCorp LP.; 2015.

[35] International Agency for Research on Cancer, Editor. IARC handbooks on cancer prevention, tobacco control, volume 12, methods for evaluating tobacco control policies: represents the views and opinions of an IARC working group on methods for evaluating tobacco control policies which met in Lyon, France, 12–19 march 2007. Lyon: International Agency for Research on Cancer; 2008. 459 p.

[36] Sassi F, Belloni A, Mirelman AJ, Suhrcke M, Thomas A, Salti N (2018). Equity impacts of price policies to promote healthy behaviours. Lancet Lond Engl..

[37] Zhang F, Wagner AK, Ross-Degnan D (2011). Simulation-based power calculation for designing interrupted time series analyses of health policy interventions. J Clin Epidemiol.

[38] Zhong Y, Auchincloss AH, Lee BK, Kanter GP (2018). The short-term impacts of the Philadelphia beverage tax on beverage consumption. Am J Prev Med.

[39] Jandoc R, Burden AM, Mamdani M, Lévesque LE, Cadarette SM (2015). Interrupted time series analysis in drug utilization research is increasing: systematic review and recommendations. J Clin Epidemiol.

[40] Donaldson E. Advocating for sugar sweetened beverage taxation [internet]: Johns Hopkins University Press; 2015. Available from: https://www.jhsph.edu/departments/health-behavior-and-society/_pdf/Advocating_For_Sugar_Sweetened_Beverage_Taxation.pdf

[41] Healthy Caribbean Coalition. The implementation of taxation on sugar sweetened beverages by the Government of Barbados: A civil society perspective [Internet]. Healthy Caribbean Coalition; 2016 Jul. Available from: http://www.healthycaribbean.org/wp-content/uploads/2016/07/HCC-SSB-Brief-2016.pdf

[42] Kostova D, Chaloupka FJ, Frieden TR, Henning K, Paul J, Osewe PL (2017). Noncommunicable disease risk factors in developing countries: policy perspectives. Prev Med.

[43] Powell LM, Chaloupka FJ (2009). Food prices and obesity: evidence and policy implications for taxes and subsidies. Milbank Q.

