# Supplementary material for: Assessing the impact of the Barbados sugar-sweetened beverage tax on beverage sales: an observational study
Source: Int J Behav Nutr Phys Act. 2019 Jan 30;16:13. doi: 10.1186/s12966-019-0776-7 (PMC6354371; doi:10.1186/s12966-019-0776-7)
Supplement: Supplementary file 1 — A description of additional methods, descriptive data, sensitivity analyses and more detailed results tables and figures (DOCX 70 kb) [file 12966_2019_776_MOESM1_ESM.docx]

**Supporting Information**

1. Supplement Table 1: Beverage Categories
2. Supplement Table 2: Summary of demographic data, Barbados and Trinidad & Tobago
3. Supplement Table 3: Absolute and Relative Weekly Effect Estimates at specific time points
4. Supplement Table 4: Mean post-tax absolute and relative effects, controlled with Trinidad and Tobago
5. Supplement Table 5: Mean post-tax absolute and relative effects, controlled with vinegar
6. Supplement Table 6: Absolute and Relative Weekly Effect Estimates at specific time points, by price tertile
7. Supplement Table 7: Post-tax trend estimates by price tertile, test for significance between tertile trends at 5%
8. Supplement Table 8: Coefficients from all Models
9. Supplement Text 1: Sales change analysis
10. Supplement Text 2: Sensitivity analyses with Trinidad & Tobago and vinegar as controls
11. Supplement Text 3: Price-tertile analysis
12. Supplement Box 1: Quality Criteria for ITS Designs (from Ramsay 2003)

**Supplement Table 1: Beverage Categories**

| Overall Categories | Sub-categories | Detailed Product Categories |
| --- | --- | --- |
| SSBs (taxed) | Carbonated SSBs | Sodas, sport drinks, energy drinks |
|  | Other SSBs | Sweetened juice drinks, malt beverages, sweetened flavored waters, other sweetened drinks |
| Non-SSBs (untaxed) | Water | Bottled waters |
|  | Other non-SSBs | Unsweetened juices, drinks with only artificial sweeteners (diet soda, diet energy and sports drinks), unsweetened flavoured waters, other unsweetened drinks |

Note: We did not consider alcoholic beverages, dairy, seasoning juices (i.e. lemon or lime juice), concentrates, syrups, or powders used to make drinks in this analysis.

**Supplement Table 2: Summary of demographic data, Barbados and Trinidad & Tobago**

| Indicator | Barbados | Trinidad |
| --- | --- | --- |
| Population (2016)^1^ | 284,996 | 1,364,962 |
| Percent Population ages 0-14^2^ | 19.7% | 20.6% |
| Percent Population ages 15-64^2^ | 67.3% | 70.5% |
| Percent Population ages 65+^2^ | 13.0% | 9.0% |
| GDP per capita, PPP (current international $, 2016)^1,3^ | 18,065 | 32,855 |
| Land area (sq. km) | 430 | 5,130 |
| Annual tourism arrivals, 2016^1^ | 632,000 | 410,000 |
| Improved water source (% of population with access, 2015)^1^ | 99.7% | 95.1% |
| Premature mortality from CVD, cancer, diabetes or CRD  (% of total, 2015^1,4^ | 16% | 26% |
| Estimated mean SSB intake, 8 oz. servings/day  (Females, ages 25-34), 2010^5^ | 4.4 | 4.7 |
| Estimated mean SSB, 8 oz. servings/day (Males, ages 25-34), 2010^5^ | 4.8 | 5.1 |
| Estimated prevalence of overweight and obesity (Females, 2013)^6^ | 69.9% | 66.1% |
| Estimated prevalence of overweight and obesity (Males, 2013)^6^ | 57.5% | 55.5% |

^1^Data from World Bank

^2^Data from 2012 Revision of the World Population Prospects

^3^GDP= Gross Domestic Product, PPP= Purchasing Power Parity

^4^ CVD=cardiovascular disease, CRD Chronic respiratory disease

^5^Data from Singh GM, Micha R, Khatibzadeh S, Shi P, Lim S, Andrews KG, et al. Global, Regional, and National Consumption of Sugar-Sweetened Beverages, Fruit Juices, and Milk: A Systematic Assessment of Beverage Intake in 187 Countries. PLOS ONE. 2015 Aug 5;10(8):e0124845. [1]

^6^Data from Ng M, Fleming T, Robinson M, Thomson B, Graetz N, Margono C, et al. Global, regional, and national prevalence of overweight and obesity in children and adults during 1980–2013: a systematic analysis for the Global Burden of Disease Study 2013. The Lancet [Internet]. 2014 May [cited 2014 Aug 8]; Available from: http://www.thelancet.com/journals/lancet/article/PIIS0140-6736(14)60460-8/fulltext [2]

**Supplement Table 3: Absolute and Relative Weekly Effect Estimates at specific time points (first full month post-tax, 6- and 12-months thereafter)**

**Supplement Table 4: Mean post-tax absolute and relative effects, controlled with Trinidad and Tobago**

**Supplement Table 5: Mean post-tax absolute and relative effects, controlled with vinegar**

**Supplement Table 6: Absolute and Relative Weekly Effect Estimates at specific time points, by price tertile**

**Supplement Table 7: Post-tax trend estimates by price tertile, test for significance between tertile trends at 5%**

**Supplement Table 8: Coefficients from all Models**

**Supplement Text 1: Sales change analysis**

Other SSB tax evaluations have relied on commercial household purchase panels, but these data are not available in Barbados. Given that the tax was implemented just three months after announcement, there was little opportunity to collect primary pre-tax data. Instead, we rely on electronic point of sale data from a major grocery store chain. According to a report by McKinsey & Company, this grocery store chain has 32% grocery store market share in Barbados [personal communication].

Data on the number of tourist arrivals per month were extracted from Trading Economics for Barbados and from the Tourism Statistics Office for Trinidad and Tobago. The monthly Consumer Price Index (CPI) was extracted from the International Monetary Fund (IMF) World Economic Outlook database. Additional descriptive data about Barbados and Trinidad and Tobago were extracted from the World Bank Open Data online database for the most recent year available, and from relevant SSB and obesity studies.

We used an ordinary least squares regression model, assuming a normally distributed outcome, separately for SSBs and non-SSBs:

$${mL/capita}_{wy}\sim\beta_{M}M_{M}+\beta_{M}{Tourism}_{M}+\beta_{Mc}{Inflation}_{M}+\beta_{w}{Holidays}_{w}+\beta_{wy}{Trend}_{wy}+\beta_{w}y{Tax}_{wy}+\beta_{wy}{TaxTrend}_{wy}+\beta_{w-1,y}{Residual}_{w-1,y}+\varepsilon_{wy}$$

where *M* denotes the vector of month indicators (1-11), *Tourism* denotes country-month specific tourism arrivals, *Inflation* denotes the country-month specific consumer price index (CPI), *Holidays* denotes the vector of indicators for Crop Over, Easter, and Christmas, *Trend* denotes the overall week-year linear trend, *Tax* denotes an indicator for the period after tax implementation, and *TaxTrend* denotes the linear week-year trend after tax implementation. Residual denotes the 1-week lag of the residual, included to address potential autocorrelation, and $\varepsilon$ represents the error term.

We tested additional specifications for seasonality, including a linear trend and two cosine and sine fourier functions, and used the Aikake Information Criterion (AIC) to assess model fit. On this basis, monthly indicators were selected as appropriate given model fit and flexibility, and because of the relatively large time series (200 observations).

To estimate average post-tax effects with uncertainty, we used the following steps:

1. Calculated the absolute difference between the estimated and counterfactual scenarios for each post-tax week
2. Estimated the average absolute difference between the estimated and counterfactual scenarios taking into account uncertainty in each estimate (using metan command in STATA)
3. Estimated the counterfactual estimate for each post-tax week
4. Estimated the average counterfactual estimate taking into account uncertainty in each estimate (using metan command in STATA)
5. Simulated 1000 estimates of the average absolute difference between the estimate and the counterfactual and of the average counterfactual, and took the ratio of each of these combinations (average absolute difference_1_/average absolute counterfactual_1,_ average absolute difference_2_/average absolute counterfactual_2_… average absolute difference_1,000_/average absolute counterfactual_1,000_)
6. Ordered the ratios by size and took the 25^th^, 500^th^ and 975^th^ observations for the estimate and 95% CI to estimate relative change (presented in % by multiplying by 100)

**Supplement Text 2: Sensitivity analyses with Trinidad & Tobago and vinegar as controls**

First, we used data from the same grocery store chain in Trinidad &Tobago, a neighbouring country where an SSB tax has not been implemented. While it would be ideal to select a comparison group with high exchangeability with the intervention group, at the country level it is inherently difficult to find a fully exchangeable comparison country[8]. In Supplement Table 2 we present key country-specific summary measures, providing reassurance that comparisons between Barbados and Trinidad & Tobago are justified, particularly in terms of similar population demographics, SSB consumption and obesity levels.

The data from Trinidad & Tobago represent the same outcomes (SSB and non-SSB sales in the same grocery store chain) in a population without the intervention [9]. This allows us to partially assess the extent to which exogenous factors, which may have coincided with the implementation of the Barbados SSB tax, possibly influenced sales of SSBs or non-SSBs. To offset differences in sales driven by differences in underlying population, we standardized the outcome of interest to milliliter sold per capita per week (recognizing that this does not adjust for the relative market share that this grocery store chain has in Trinidad & Tobago compared to in Barbados, as these data were not available). We included country-specific consumer price indices to control for inflation.

As a second sensitivity analysis, we used a non-beverage product (vinegar) to assess a different outcome in the same population and test whether there may have been some factor that influenced sales at this grocery store chain. The introduction of the SSB tax coincided with the removal of roughly 50% of items that were previously exempt from value added tax (VAT) (vinegar was already subject to VAT) [10]. The Minister of Health announced that removed items had been reviewed and identified as “more sophisticated things than would have been described as basic,” but that “for every item that has been taken out, a nutritious equivalent substitute remains in the basket” [10]. Items that were removed from the basket were subjected to the standard 17.5% VAT rate. We attempted to address this concern by assessing whether there was a change in sales of a product that should not have been affected by the SSB tax but could have been affected by changing overall prices (and therefore, changing disposable income.)

The decision to use vinegar was pragmatic (based on data availability), although it is possible that vinegar may be somewhat less responsive to immediate changes in price due to a longer lag between purchases. Other planned evaluations intend to use personal toiletries as a similar type of control product[11].

We used an ordinary least squares model, assuming a normally distributed outcome, separately for SSBs and non-SSBs:

$${mL/capita}_{wyc}\sim\beta_{m}M_{m}+{\beta_{mc}{M*Control}_{mc}+\beta}_{myc}{Tourism}_{myc}+\beta_{myc}{Inflation}_{myc}+\beta_{wyc}{Holidays}_{wyc}+\beta_{wy}{Trend}_{wy}+\beta_{wy}{Tax}_{wy}+\beta_{wy}{TaxTrend}_{wy}+\beta_{c}{Control}_{c}{+\beta_{wyc}{Trend*Control}_{wyc}+\beta_{wyc}{Tax*Control}_{wyc}+\beta_{wyc}{TaxTrend*Control}_{wyc}+\beta}_{w-1,yc}{Residual}_{w-1,yc}+\varepsilon_{wyc}$$

where *M* denotes the vector of month indicators (1-11), *M*Control* denotes the control-specific vector of month indicators, *Tourism* denotes tourism arrivals, *Inflation* denotes the consumer price index (CPI) and *Holidays* denotes the vector of indicators for Crop Over (Barbados only), Carnival (Trinidad & Tobago only) Easter and Christmas. The subscript *w* corresponds to week-specific variables (1-52), *m* corresponds to month-specific variables (1-12), *y* corresponds to year-specific variables (2013-2016), *wy* corresponds to week-year specific variables (1-200), *my* corresponds to month-year specific variables (1-50) and *c* corresponds to control-specific variables. *Trend* denotes the overall week-year linear trend, *Tax* denotes an indicator for the period after tax implementation, and *TaxTrend* denotes the linear week-year trend after tax implementation. *Control* denotes the indicator for the control (either country or vinegar), *TrendControl* denotes the interaction between the control and the overall week-year linear trend, *TaxControl* denotes the interaction between the control and the tax indicator, and *TaxTrendControl* denotes the interaction between the control and the post-tax linear trend. *Residual* denotes the 1-week lag of the residual, included to address potential autocorrelation, and $\varepsilon$ represents the error term.

**Supplement Text 3: Price-tertile analysis**

We defined price tertile accordingly:

1. Within each country-category, we estimated the mean price of each product over the whole period (to capture products that were introduced after the tax)
2. We took the total litres sold per week of each product over the whole period
3. We sorted products (within their country-category) by mean price, and then identified price tertile cut-off points at 33% and 66% of the total litres sold within that country-category.

We then used an ordinary least squares model, assuming a normally distributed outcome, separately for SSBs and non-SSBs:

$${mL/capita}_{wy}\sim\beta_{M}M_{m}+\beta_{my}{Tourism}_{my}+\beta_{my}{Inflation}_{my}+\beta_{w}{Holidays}_{w}+\beta_{wy}{Trend}_{wy}+\beta_{w}y{Tax}_{wy}+\beta_{wy}{TaxTrend}_{wy}+\beta_{wy}{Tertile}_{wy}+\beta_{wy}{TrendTertile}_{wy}+\beta_{w}y{TaxTertile}_{wy}+\beta_{wy}{TaxTrendTertile}_{wy}+\beta_{w-1,y}{Residual}_{w-1,y}+\varepsilon_{wy}$$

where *M* denotes the vector of month indicators (1-11), *Tourism* denotes country-month specific tourism arrivals, *Inflation* denotes the country-month specific consumer price index (CPI) and *Holidays* denotes the vector of indicators for Crop Over, Easter and Christmas. The subscript *w* corresponds to week-specific variables (1-52), *m* corresponds to month-specific variables (1-12), *y* corresponds to year-specific variables (2013-2016), *wy* corresponds to week-year specific variables (1-200) and *my* corresponds to month-year specific variables (1-50). *Trend* denotes the overall week-year linear trend, *Tax* denotes an indicator for the period after tax implementation, and *TaxTrend* denotes the linear week-year trend after tax implementation. Residual denotes the 1-week lag of the residual, included to address potential autocorrelation, and $\varepsilon$ represents the error term.

*Tertile* denotes an indicator for each tertile (with the first tertile set as the reference category), *TrendTertile* denotes the interaction between the tertile indicators and the overall week-year linear trend, *TaxTertile* denotes the interaction between the tertile indicators and the tax indicator, and *TaxTrendTertile* denotes the interaction between the tertile indicators and the linear week-year trend after tax implementation.

As a sensitivity analysis, we categorized drinks using only pre-tax prices, but this did not substantially change the pattern of results.

**Supplement Box 1: Quality Criteria for ITS Designs (from Ramsay 2003)**

1. Intervention occurred independently of other changes over time

The intervention occurred at the same time as the change in VAT-exempt products was implemented. However, this was tested as a potential time-varying confounder by controlling the grocery store analysis with a non-beverage product (vinegar).

2. Intervention was unlikely to affect data collection

The intervention itself was unlikely to affect data collection because we used routine data sources that were used for different purposes and did not change following the intervention.

3. The primary outcome was assessed blindly or was measured objectively

The primary outcome variables (sales of SSBs and non-SSBs) were recorded objectively as part of an electronic point of sale system and a national import/export ledger.

4. The primary outcome was reliable or was measured objectively

As above.

5. The composition of the data set at each time point covered at least 80% of the total number of

participants in the study

The grocery store dataset covered 100% of sales made over the study period at this grocery store chain. We acknowledge that the grocery store chain has been estimated to account for 32% of the total grocery store market share in Barbados [personal communication].

6. The shape of the intervention effect was prespeciﬁed

We pre-specified a slope and intercept change following implementation of the tax, based on results from other SSB tax evaluation studies.

7. A rationale for the number and spacing of data points was described

We used all available data from January 2013 on. Data were aggregated by week, so it was not possible to conduct an analysis at the daily level. Since a competitor opened a new store in Barbados in November 2016, we limited our analysis of the grocery store chain data to the period ending on October 31, 2016. With 141 pre-tax observations and 59 post-tax observations for the grocery store analysis we had a long and balanced time series which has been shown to add strength to the ITS design [12].

8. The study was analyzed appropriately using time series techniques

We used segmented time series regression models to analyze the data and serial correlation was adjusted for using a lagged residual.
